# Stroke in a patient of anaphylaxis—a case report and brief review

**DOI:** 10.1186/s12245-023-00548-3

**Published:** 2023-10-17

**Authors:** Takshak Shankar, Nagasubramanyam Vempalli, Reshma Asokan, Aadya Pillai, D. J. Lalneiruol Infimate

**Affiliations:** 1 Department of Emergency Medicine, All India Institute of Medical Sciences, Rishikesh, India; 2 Department of Emergency Medicine, Mahatma Gandhi Medical College and Research Institute, Puducherry, India; 3 Baby Memorial Hospital, Kannur, India; 4 Department of Emergency Medicine, All India Institute of Medical Sciences, New Delhi, India

**Keywords:** Anaphylaxis, Stroke, Intravenous contrast media

## Abstract

**Background:**

The use of nonionic low-osmolar contrast media has significantly reduced the risk of hypersensitivity reactions. Despite this, severe reactions continue to occur unpredictably. An ischemic stroke in the setting of anaphylaxis is extremely rare.

**Case report:**

A 64-year-old male with no prior allergies went into anaphylactic shock following the administration of iohexol which improved after treatment. He later developed a multi-territorial ischemic stroke.

**Conclusion:**

An ischemic stroke in the setting of an anaphylaxis is a rare occurrence, which can be attributed to multiple factors in our patient.

## Background

Intravenous contrast media (ICM) causing hypersensitivity has reduced tremendously since the widespread use of nonionic low-osmolar contrast media [1]. The majority of these are nonallergic reactions, and a smaller fraction is IgE-mediated immune allergic reaction [2]. Several of these mast cell mediators are involved in coagulation and fibrinolysis pathways depending on their relative release, resulting in a disturbance of hemostasis [3].

The concurrent occurrence of acute coronary syndrome in the setting of hypersensitivity reaction following mast cell and platelet activation, and other interrelated inflammatory cells, has been widely discussed in the literature called as the Kounis syndrome (KS). KS is pan-arterial, affecting multisystem and multi-organ, namely coronary, mesenteric, and cerebral arteries [4]. Kounis-like syndrome affecting the cerebral vasculature has been described in individuals with mast cell activation disorders presenting as transient ischemic attack (TIA) [5]. We are presenting a unique case where iohexol contrast injection has precipitated anaphylaxis in a patient with no prior contrast exposure and resulted in multi-territorial embolic stroke.

## Case report

A 64-year-old gentleman presented to the emergency department complaining of sudden onset shortness of breath following intravenous contrast dye injection 30 min back. The patient had a history of painless hematuria for the last 15 days and was undergoing computed tomography-urography for evaluation. He was a reformed smoker for the last 10 years with no known comorbidities or previous allergies. He was not on any regular medications. He was being worked up for his painless hematuria and had no exposure to radiocontrast media earlier. Shortly after the intravenous administration of 80 mL of iohexol 350 milligrams (mg) iodine per milliliters, he developed uneasiness, profuse sweating, generalized itching, and difficulty breathing. He was administered hydrocortisone 200 mg intravenously by the radiologist and was shifted to the emergency immediately.

**Fig. 1:**
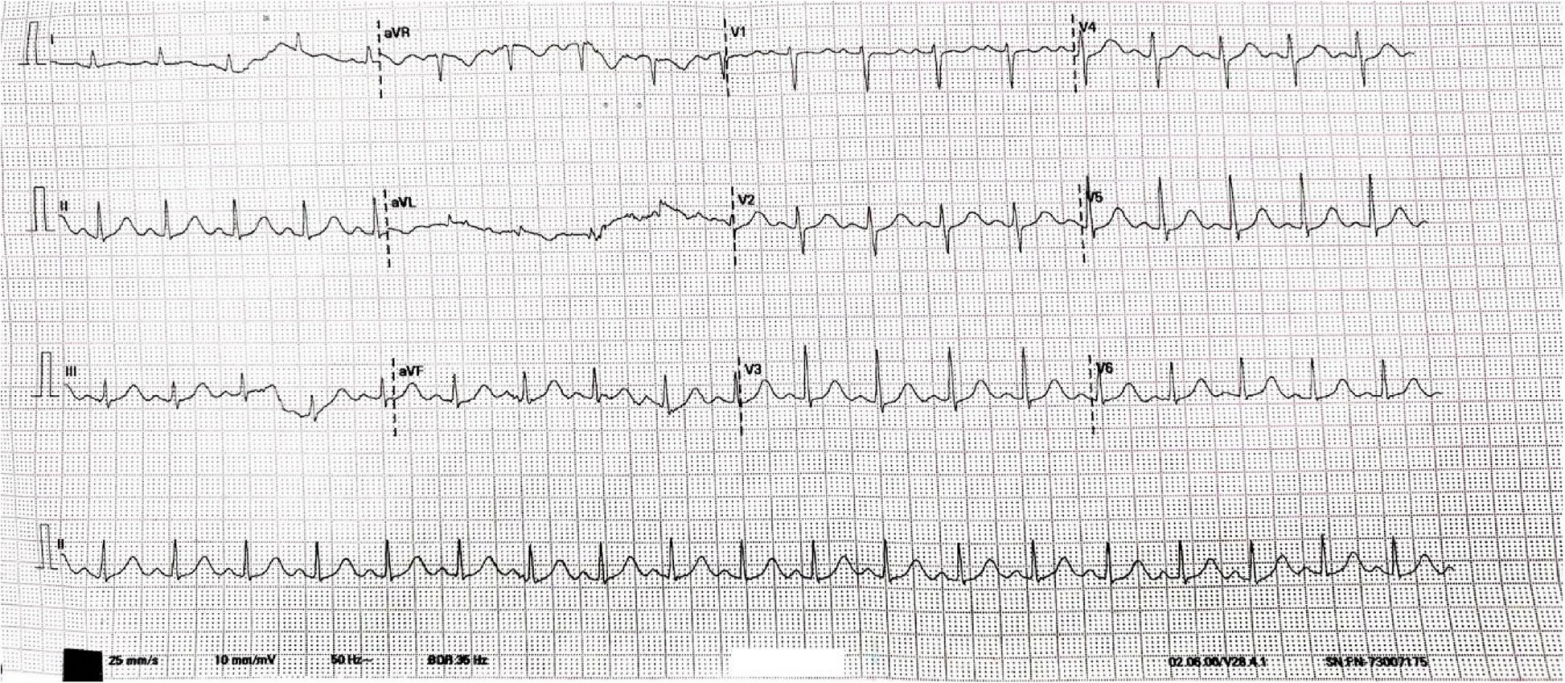
Electrocardiogram of the patient after bolus 1 L showing sinus tachycardia

On arrival, he was conscious, oriented, had generalized blanchable erythema, and was in significant respiratory distress. He had a pulse rate (PR) of 150 bpm, blood pressure (BP) of 82/54 millimeters of mercury (mm Hg), oxygen saturation (SpO_2_) of 77% on room air, and respiratory rate (RR) of 35 per minute. On auscultation, bilateral diffuse wheeze was present along with crepitations. The rest of the systemic examination was within normal limits. The venous blood gas on arrival showed a potential of hydrogen (pH) of 7.275 [reference range: 7.31–7.41], partial pressure of carbon dioxide (pCO2) of 19.3 mmHg [reference range: 41–51], partial pressure of oxygen (pO2) of 31.5 mmHg [reference range: 30–40], bicarbonate (HCO3) of 9.1 millimoles per liter (mmol/L) [reference range: 23–29], and lactate of 13.5 mmol/L [reference range: < 1.2]. Electrocardiogram (ECG) showed sinus tachycardia. He was immediately put on oxygen at 15 L/min via a non-rebreather mask and administered intravenous fluids, adrenaline 0.5 mg intramuscularly, diphenhydramine 50 mg intravenously, and ranitidine 50 mg intravenously considering the possibility of an anaphylactic reaction. His clinical condition improved considerably to a PR of 110 bpm, BP of 100/70 mm Hg, SpO_2_ of 100% on 5 L of oxygen support, and RR—24 per minute over the next half an hour. However, he developed sudden onset slurring of speech associated with deviation of angle of the mouth to the left side and slurring of speech and weakness of the right side of the body. On examination, he was conscious but not oriented to time, place, and person. CNS examination was significant for decreased power of 4/5 in both right upper and lower limbs. His plantar reflex was mute on the right side and flexor on the left side. ECG showed sinus tachycardia with no dynamic ST-T changes (Fig. 1).

**Fig. 2:**
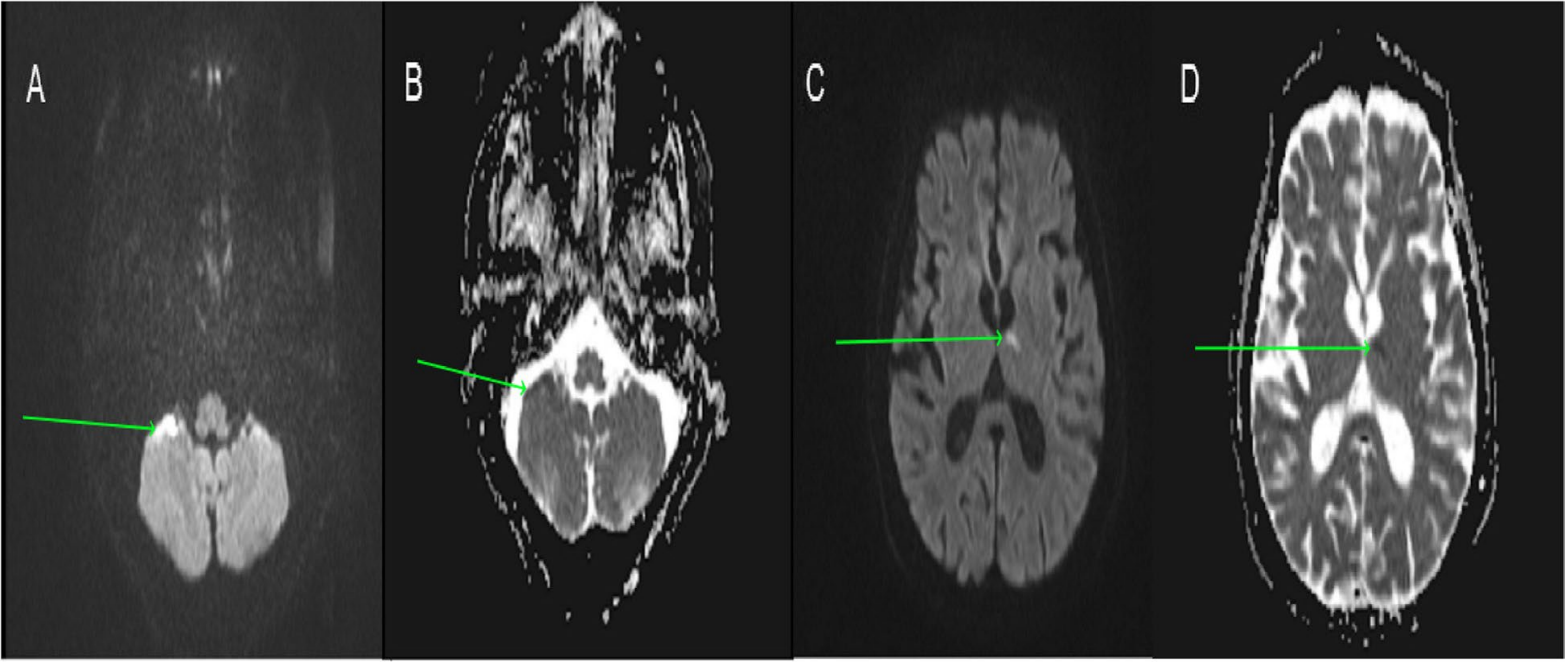
**A** Axial section. Magnetic resonance imaging of the brain showing diffusion restriction in right cerebellar hemisphere (green arrow). **B** Axial section magnetic resonance imaging of the brain showing low apparent diffusion coefficient values in right cerebellar hemisphere (green arrow). **C** Axial section magnetic resonance imaging of the brain showing diffusion restriction in left thalamus (green arrow). **D** Axial section magnetic resonance imaging of the brain showing low apparent diffusion coefficient values in left thalamus (green arrow)

The patient was shifted for a magnetic resonance imaging (MRI) of the brain immediately. It showed multifocal areas of diffusion restriction in left thalamus, left medial temporal lobe, left insular cortex, bilateral putamen, right middle cerebellar peduncle, right cerebellar hemisphere, and right half of pons, and an impression of multi-territorial acute infarcts was made (Fig. 2).

Owing to active hematuria, the patient was not thrombolyzed and was put on only supportive management, following which his hemodynamic parameters improved.

The investigations on arrival to the emergency are summarized in Table 1.

**Table 1:** Investigations of the patient

Parameters	Emergency arrival
Hemoglobin (grams per deciliter)	3.259 [reference range:13–17]
Platelet count (per cubic millimeter)	375100 [reference range: 150,000–450,000]
INR	1.06 [reference range: 0.90–1.10]
SARS CoV-2 rRT-PCR	Negative
TSH (micro international units per milliliter)	3.39 [reference range: 0.35–5.5]
FT4 (nanogram per milliliter)	1.14 [reference range: 0.89–1.76]
Serial troponin I	Negative
2D-ECHO	Left ventricular ejection fraction—55–60%, no regional wall motion abnormality, right atrium and ventricle normal, no clot, no vegetation

*N/L/M* neutrophils/lymphocytes/monocytes *INR*, international normalized ratio *SARS-CoV-2 rRT-PCR* severe acute respiratory syndrome coronavirus-2 real-time reverse transcriptase polymerase chain reaction *2D-ECHO* two-dimensional echocardiography *TSH*, thyroid-stimulating hormone *FT4*, free thyroxine

The patient was then shifted to the intensive care unit. Holter monitoring done during hospital stay was normal. He later underwent radical cystectomy with ileal conduit during his hospital stay for non-muscle invasive bladder cancer, as it was endoscopically unmanageable. After the resolution of hematuria, he was started on tab aspirin 150 mg once daily. He was discharged after a month in a stable condition with residual weakness of right upper and lower limb (power 4/5) and slurring of speech. There was no visual impairment. After 6-month follow-up, the patient continued to have some slurring of speech, with complete resolution of the limb weakness.

## Discussion

The hypersensitivity to intravenous contrast media has been broadly classified as immediate reactions (IR) and non-immediate reactions (NIR). The immediate reactions occur within 1 h and can be IgE mediated or non-IgE mediated [1, 6]. Non-immediate reaction occurs within hours or even days and has T-cell involvement and lymphocyte proliferation. Since this patient developed an allergic reaction within 1 h of contrast instillation, he had an immediate reaction. It was associated with urticaria, flushing, bronchoconstriction, and hypotension, suggesting anaphylaxis. The incidence of immediate hypersensitivity reaction to nonionic contrast lies between 0.01 and 0.04% in severe cases and up to 3% in mild cases. Risk factors include previous history of reaction to contrast media, history of asthma and atopy, heart diseases, renal diseases, thyroid abnormalities, medications like metformin, and beta blockers and in the elderly (greater chance of severe reaction) [7].

Usually, the brain does not suffer from allergic reactions since IgE cannot cross the blood-brain barrier even though mast cells (MC) are present in the brain. But a majority of contrast medium allergies happen even in the absence of previous exposure and are non-IgE mediated. Symptoms are due to release of inflammatory mediators from mast cells either directly by non-specific binding of contrast media particles to membrane receptors or indirectly by complement-kinin activation (C5a) [2].

There are case reports in the literature on contrast medium hypersensitivity causing Kounis syndrome (KS) [8, 9]. There are three variants of Kounis syndrome—vasospastic allergic angina, allergic myocardial infarction due to plaque rupture, and stent thrombosis with occluding thrombus infiltrated by eosinophils and mast cells [4]. Similarly, vasospasm causing symptoms in other organs like transient ischemic attacks (TIA) in patients of known mast cell activation disorders and allergic reactions causing abdominal symptoms due to vasospasm of splanchnic artery or embolism causing bowel ischemia has also been described [5, 10]. But anaphylactic reaction to contrast medium resulting in multi-territorial cerebral infarction has not been reported before to the best of our knowledge.

When activated by C5a complement, mast cells switch from a profibrinolytic phenotype in the resting state to a prothrombotic phenotype by expressing plasminogen activator inhibitor 1 (PAI-1) [11]. Prothrombotic mechanisms include triggering platelet activation and aggregation by PAF, activation of factor XII by heparin, activation of fibrinogen, factors XII and XIII by proteases like tryptase and chymase, secretion of vWF and factor VIII, and promoting the expression and release of tissue factor and plasminogen activator inhibitor into circulation. Mast cells also produce extracellular traps, the presence of which has been reported in coronary thrombi formation. A case series had pointed out the risk of anaphylaxis in causing secondary thrombosis, and it is highest in the first 72 h [12]. The patient also had severe anemia, probably due to chronic blood loss in the form of hematuria. Chronic blood loss can also stimulate erythrocytes and platelets, resulting in thrombocytosis as well [13, 14].

PAF cause a decrease in myocardial contractility, which result in reduced cardiac output. There is also loss of intravascular component due to increased vascular permeability and vasodilation in the systemic circulation. The anaphylactic mediators—histamine, chymase, and leukotrienes, along with PAF, can induce cerebral artery spasm too. Hence, the reduction in cerebral blood flow due to anaphylactic shock is greater than that due to mere arterial hypotension [15].

This patient was diagnosed with a noninvasive bladder cancer later which caused the hematuria in this patient. But the association with thromboembolism is negligible in noninvasive bladder cancer. The risk increases only in muscle-invasive bladder cancer (up to 6%) and up to twofold times if the patient has undergone radical cystectomy [16].

Thus, a prothrombotic environment with acute reduction in cerebral blood flow due to anaphylaxis is hypothesized to be the causative factor behind stroke in our patient and makes this a unique case.

## Conclusion

Severe allergic reactions to contrast medium still occur unpredictably in some patients even with the usage of nonionic low-osmolar contrast media. It is crucial to recognize severe anaphylactic reactions early, as they can be life-threatening. Although an ischemic stroke is extremely rare in the setting of anaphylaxis, its timely recognition and management can change patient outcomes considerably.

## Data Availability

Not applicable

## Abbreviations

ICM: Intravenous contrast media
KS: Kounis syndrome
mg: Milligrams
PR: Pulse rate
BP: Blood pressure
SpO2: Saturation of peripheral oxygen
RR: Respiratory rate
pH: Potential of hydrogen
pCO2: Partial pressure of carbon dioxide
pO2: Partial pressure of oxygen
mmol/L: Millimoles per liter
ECG: Electrocardiogram
MRI: Magnetic resonance imaging
N/L/M: Neutrophils/lymphocytes/monocytes
INR: International normalized ratio
SARS-CoV-2 rRT-PCR: Severe acute respiratory syndrome coronavirus-2 real-time reverse transcriptase polymerase chain reaction
2D-ECHO: Two-dimensional echocardiography
TSH: Thyroid-stimulating hormone
FT4: Free thyroxine
WAO: World Allergy Organization
IgE: Immunoglobulin E
MC: Mast cells
TIA: Transient ischemic attacks
